# Mapping Clinical Barriers and Evidence‐Based Implementation Strategies in Low‐to‐Middle Income Countries (LMICs)

**DOI:** 10.1111/wvn.12503

**Published:** 2021-05-10

**Authors:** Ashley Whitehorn, Liang Fu, Kylie Porritt, Lucylynn Lizarondo, Matthew Stephenson, Tania Marin, Aye Aye Gyi, Kim Dell, Alex Mignone, Craig Lockwood

**Affiliations:** ^1^ JBI The University of Adelaide Adelaide SA Australia; ^2^ Department of Nursing Jinhua Municipal Central Hospital Jinhua Zhejiang Province China; ^3^ School of Nursing Fudan University Shanghai China

**Keywords:** implementation, knowledge translation, evidence‐based practice, barriers, strategies, low‐to‐middle income countries, LMIC

## Abstract

**Background:**

Low‐to‐middle income countries (LMICs) experience a high burden of disease from both non‐communicable and communicable diseases. Addressing these public health concerns requires effective implementation strategies and localization of translation of knowledge into practice.

**Aim:**

To identify and categorize barriers and strategies to evidence implementation in LMICs from published evidence implementation studies.

**Methods:**

A descriptive analysis of key characteristics of evidence implementation projects completed as part of a 6‐month, multi‐phase, intensive evidence‐based clinical fellowship program, conducted in LMICs and published in the JBI Database of Systematic Reviews and Implementation Reports was undertaken. Barriers were identified and categorized to the Donabedian dimensions of care (structure, process, and outcome), and strategies were mapped to the Cochrane effective practice and organization of care taxonomy.

**Results:**

A total of 60 implementation projects reporting 58 evidence‐based clinical audit topics from LMICs were published between 2010 and 2018. The projects included diverse populations and were predominantly conducted in tertiary care settings. A total of 279 barriers to implementation were identified. The most frequently identified groupings of barriers were process‐related and associated predominantly with staff knowledge. A total of 565 strategies were used across all projects, with every project incorporating more than one strategy to address barriers to implementation of evidence‐based practice; most strategies were categorized as educational meetings for healthcare workers.

**Linking Evidence to Action:**

Context‐specific strategies are required for successful evidence implementation in LMICs, and a number of common barriers can be addressed using locally available, low‐cost resources. Education for healthcare workers in LMICs is an effective awareness‐raising, workplace culture, and practice‐transforming strategy for evidence implementation.

## BACKGROUND

Low‐to‐middle income countries (LMICs) experience a high burden of disease from both non‐communicable and communicable diseases (Ojo et al., 2019). Addressing these public health concerns requires effective implementation strategies and localization of translation of knowledge into practice (Edwards, Zweigenthal, & Olivier, 2019). In LMICs, where resources are scarce and burden of disease is high, justification to intervene in healthcare practice must be based on high‐quality, evidence‐based findings (Edwards et al., 2019). However, despite a growing body of research to inform clinical decision‐making that considers the best available evidence (Jordan, Lockwood, Munn, & Aromataris, 2018, 2019), the uptake of research findings into clinical practice remains inadequate (Pinnock et al., 2017; Santesso & Tugwell, 2006).

There are several features unique to LMICs that add an additional layer of complexity to the implementation of evidence‐based practice (EBP), such as a high burden of disease and extreme human and resource shortages (Puchalski Ritchie et al., 2016). In countries faced with these circumstances, the need to implement effective and efficient healthcare strategies becomes even more important. To bridge the knowledge‐to‐practice gap and ensure effective healthcare practices are applied, a locally designed implementation plan that considers context‐specific barriers and strategies and identifies implementation resources is imperative (Puchalski Ritchie et al., 2016).

This paper contributes to the global knowledge base on barriers toward implementing EBP in LMICs by evaluating the published results of JBI implementation studies conducted in LMIC settings. These studies rely upon a standardized model of education aligned with local, context‐specific implementation plans that address both the issues in practice and the knowledge and skill needs of clinicians who may be expected to lead or sustain a change process but are rarely equipped, supported, or enabled with the skills and knowledge to succeed.

The JBI Evidence‐Based Clinical Fellowship Program (EBCFP) is a 6‐month, multi‐phase, intensive program that equips clinicians with the necessary leadership and practice skills required for implementing evidence into practice. The EBCFP is available to clinicians, managers, policymakers, and quality managers from all over the world with an interest in evidence implementation in day‐to‐day clinical practice. It commences with a 1‐week residency program on the fundamentals of evidence‐based health care, change management, and clinical leadership, with participants provided with ongoing access to evidence‐based resources, facilitation, and mentorship in practice change (Lizarondo, Lockwood, & McArthur, 2019).

As part of the program, participants undertake a three‐phased evidence implementation project using audit and feedback to facilitate practice change—an effective strategy for implementing evidence into practice (Ivers et al., 2012). Upon completion of the first week, participants return to their practice setting to create a local project team and commence baseline data collection to establish current compliance with best practice recommendations; this step is referred to as phase one of the project. The second phase occurs after baseline data collection. Project teams collaborate with their stakeholders to undertake a situational analysis, identify barriers and enablers to compliance, and develop an action plan to implement strategies that address the identified barriers. The JBI approach to planning for practice change with a focus on sustainable strategies is entitled JBI‐GRiP (Getting Research into Practice), which is built into a software program (Practical Application of Clinical Evidence System [PACES]) that each participant uses to support the process (Lizarondo et al., 2019).

The JBI‐GRiP guides situational analysis, that is, participants identify and align facilitator and barrier reduction strategies to audit criteria where compliance was suboptimal (optimal being agreed upon by the stakeholders). In JBI‐GRiP, once the barriers are identified, strategies and facilitators to address each barrier can be determined. After barrier identification and strategy determination, the team can then pinpoint available resources or consider additional resources that may be required. Resources such as education or training materials can be developed or sought after externally then localized. Strategies to implement resources are then noted in JBI‐GRiP to close the loop.

Undertaking a situational analysis using the JBI‐GRiP process enables transparent planning and reporting of the resources and strategies used to address specific barriers. The use of situational analysis within local contexts with key staff and stakeholders ensures that the implementation planning is aligned to the needs and priorities of the organization, and, we argue, facilitates adaptability and sustainability. This phase generally continues between 4 to 6 months depending on locally available resources and progress with implementing change. The data produced in this second phase via situational analysis (an exemplar GRiP of common barriers and strategies is shown in Table S1) is published in completed implementation reports; these data form the focus of this project.

The final phase is a post‐implementation evaluation using the same audit criteria that were measured in phase one. It is this final phase that provides the comparative data of change, and as data are entered, JBI‐PACES provides live updates of changes in compliance that tell the team of the direction and extent of change.

The approach to implementation adopted in the JBI EBCFP is the mechanisms embedded in clinical audit. Quality improvement using audit and feedback is the core component guiding the collection of data and methods for facilitating engagement of key stakeholders; the theoretical underpinning that informs the audit component of the fellowship is the three dimensions of Donabedian’s (2005) quality improvement framework. Donabedian (2005) conceptualized the evaluation of care into three dimensions: structure, process, and outcome (SPO). Structure is defined as settings, qualifications, and administrative processes that guide the planning and evaluation of organizational characteristics that influence practice change; process considers the components of care delivery; and outcome considers the patient’s recovery, restoration of function, and survival (Ayanian & Markel, 2016). This framework assists in developing a deeper understanding of the barriers and facilitators when changing practice within a quality improvement framework (Ayanian & Markel, 2016). As a framework, it facilitates measurement across care delivery, the organizational infrastructure and systems, and the interplay and interdependence of the many factors influencing practice change (Ivers et al., 2012). Additionally, the framework has been used in multiple settings to evaluate quality and safety in health care, including nursing (Gardner, Gardner, & O'Connell, 2014), surgery (Brownlee, Whitson, & Ibrahim, 2019), community disease prevention (Rai & Wood, 2017), and chronic disease management (Ameh, Gómez‐Olivé, Kahn, Tollman, & Klipstein‐Grobusch, 2017). The Donabedian framework can also be used to analyze the success of implementation projects, providing a valuable opportunity to map and document both the success and failures of getting research into practice.

The Cochrane Effective Practice and Organisation of Care Group (EPOC) taxonomy classifies health system interventions into categories based on their conceptual or practical similarities (Cochrane, 2015). The EPOC taxonomy consists of four domains: delivery arrangements, financial arrangements, governance arrangements, and implementation strategies. This classification is useful in facilitating the synthesis and analysis of evidence relating to implementation interventions for different practice settings. These enable a more granular, detailed level of categorization, and analysis of data than the higher level Donabedian framework, which considers broader categories, particularly in relation to organizational arrangements for delivery of care. Mapping interventions against the EPOC taxonomy assists with cross‐comparisons, the benefits of which are the facilitation of conceptual clarity in distinguishing between an implementation strategy such as audit and feedback (Slaughter, Hill, & Snelgrove‐Clarke, 2015) and an intervention such as an education program or policy change process to align with best practice recommendations. Authors have noted that the level of intervention is also difficult to classify, highlighting that the level of classification affects capacity to report accurately and reliably (Slaughter et al., 2015). These concerns are potentially reduced when using the more granular EPOC taxonomy.

For this study, data from a published cohort of implementation studies conducted in LMICs were analyzed in order to identify and categorize barriers and facilitators to EBP change in LMIC contexts.

### Aim

The aim of this study was to identify and categorize barriers and strategies to evidence implementation in LMICs from published evidence implementation studies. Specifically, the objectives were to identify barriers to compliance with best practice across topics and settings; categorize barriers to best practice identified through each individual project using the Donabedian dimensions of care (SPO); and map the implementation strategies used in different projects against the EPOC taxonomy.

## METHODS

This paper presents a descriptive analysis of key characteristics of previously published implementation projects from LMICs extracted from the JBI Database of Systematic Reviews and Implementation Reports (JBISRIR), now known as JBI Evidence Synthesis.

### Sampling

Published implementation reports were identified in the JBISRIR and selected if the project was conducted in a country classified as an LMIC according to the World Bank Criteria (Prydz & Wadhwa, 2019). All published LMIC implementation reports were included regardless of topic, geographic location, or professional lead of the study.

### Data Extraction

Each report’s citation was downloaded into EndNote X8.2 (Clarivate Analytics) and classified by country of origin. A Microsoft Excel spreadsheet was developed to capture data including the record number, author details, publication title, project topic, types of participants, keywords associated with the publication, country of origin, year of origin, and lead profession for the project. Study‐specific data also included whether the project involved multidisciplinary teams, project setting, type of service, type of department, specific audit criteria, specific barriers associated with each criterion, implementation strategies associated with each specific barrier, and the levels of compliance pre‐ and post‐implementation.

The data extraction template (available on request from the corresponding author) was piloted by one author and then reviewed with the author group. Three additional fields were added: one to classify each barrier to practice change against the Donabedian SPO model; the second to further classify each barrier against standard types of barriers within the Donabedian (SPO) primary classifications; and the third to enable coding of the implementation strategies reported in each report against the EPOC taxonomy. Data extraction was undertaken by one author and discussed with the authorship team.

### Data Analysis

Data were collected in an Excel spreadsheet, and two authors independently classified each barrier against the Donabedian SPO classification system using separate versions of the data and allocated the types of barriers. The two authors met, compared results, and resolved differences. To assess the inter‐rater reliability of data reference to the Donabedian SPO framework, a random sample of 25 observations was selected from the database. The frequency of agreements (1 = agreed) and disagreements (2 = disagreed) between the authors was tabulated, and overall agreement was determined using kappa (*k*) statistics. The following statistical assumption was applied to the interpretation of the results: *k* 0–.20 = poor; .21–.40 = fair; .41–.60 = moderate; .61–.80 = good; and .81–1.00 = excellent. Disagreements were resolved by returning to the definitions of terms for coding barriers against the SPO and discussing through to resolution. Data were then transferred to Statistical Package for Social Science Software (SPSS version 25.0) for descriptive reporting of frequencies, mean values, and simple differences between groups.

### Identifying and Classifying Barriers

Individual project barriers identified in each implementation report were extracted. Two authors independently reviewed barriers identified for each project and grouped the like barriers across all reports.

Barriers were considered any issue or problem identified through situational analysis as a contributor to low or less than satisfactory compliance and reported in the published report. Barriers only needed to be unique to the individual audit criterion, that is, any barrier could be identified multiple times from the same report or across multiple implementation reports. Definitions for alignment to the Donabedian SPO structure were adapted from the McMaster University Toolkit for Advanced Practice Nursing Data Collection (Vohra & Bryant‐Lukosius, 2009). These consisted of a triad of structure, process, and outcome constructs where structure was defined as the characteristics of the organization and the physical setting and characteristics of the staff (e.g., availability of medicines and equipment, and staff knowledge, awareness, or skill); process as the actions of the patient or client as well as the actions of the healthcare team members in delivering care (e.g., hospital referrals and defaulter tracing); and outcome as the impact of care on the state of health and events that follow (e.g., changes in patient or client knowledge, self‐care ability, the relief or management of symptoms, changes in health condition, and satisfaction with care).

### Mapping Strategies to EPOC Taxonomy

Independently from the first process, two authors further coded the strategies implemented in the projects to address the barriers against the EPOC framework. Data were coded and sorted in Excel and moved to SPSS (version 26.0) for descriptive analysis. Each strategy was aligned with an EPOC sub‐category and then mapped back to category and topic.

## RESULTS

### Demographics

The total number of participants trained in the EBCFP worldwide from 2010 to 2018 was 472; 235 of these had published implementation reports in the JBISRIR. Of these, 60 were from LMICs, with the majority occurring in 2014 (21.7%; *n* = 13) and the least in 2010 (1.7%; *n* = 1). Table 1 provides an overview of the characteristics of the 60 implementation projects.

**Table 1:** Demographic Characteristics of Low‐to‐Middle Income Countries Implementation Case Studies Published Between 2001 and 2017

Project settings	*n* (%)
**Asia**	**39 (65.0)**
China	34 (56.7)
Malaysia	2 (3.3)
Myanmar	2 (3.3)
Indonesia	1 (1.7)
**Africa**	**18 (30.0)**
Kenya	7 (11.7)
Ghana	4 (6.7)
Ethiopia	3 (5.0)
Uganda	2 (3.3)
Cameroon	2 (3.3)
Malawi	1 (1.7)
**South America**	**3 (5.0)**
Brazil	3 (5.0)
**Departments**	
Tertiary hospitals	50 (83.3)
Outpatient clinics	8 (13.3)
Community	2 (3.3)
**Lead professionals**	
Nurse	44 (73.3)
Medical doctor	6 (10.0)
Pharmacist	4 (6.7)
Public health professional	3 (5.0)
Technical quality coordinator	1 (1.7)
Lecturer of health promotion/health Education	2 (3.3)
**Team structure**	
Multidisciplinary	29 (48.3)
Single discipline	31 (51.7)

Projects in Asia (65%) were predominantly based in China (58.3%). The remaining projects were completed in Africa (30.0%) and South America (5.0%). The projects included diverse populations such as pregnant women, children, and their families; people with heart failure, cancer, and inflammatory bowel disease; surgical patients; and healthcare professionals. Projects were conducted in a variety of healthcare settings but were predominantly in tertiary hospitals. A multidisciplinary team approach was adopted in 29 (48.3%) of the projects.

All projects were based on evidence‐based audit criteria ranging from two to 13 (average of four); these audit criteria were distilled from practice recommendations informed by the best available evidence from clinical guidelines, systematic reviews, and primary research. Across projects, compliance to these criteria was 33.6% pre‐audit and 84.3% post‐audit (50.7% overall improvement).

### Identification of Barriers

A total of 58 evidence‐based audit topics were covered in the 60 implementation projects, and all were evaluated to determine the level of compliance to best practice in the project’s setting. A total of 279 barriers were identified from the 60 projects. Table 2 presents the barriers categorized by (a) knowledge, skill, and attitude; (b) resources; and (c) organizational characteristics.

**Table 2:** Identified Barriers Categorized by (a) Knowledge, Skills, and Attitude; (b) Resources; and (c) Organizational Characteristics

Barriers	*n*	(%)
**Knowledge, skills, and attitude**	**120**	**(43.0)**
Lack of knowledge	64	(22.9)
Lack of knowledge and clinical skill	21	(7.5)
Lack of communication HP to HP/HP to patient	9	(3.2)
Poor attitude	9	(3.2)
Limited patient engagement	4	(1.4)
Lack of knowledge and patient skill	3	(1.1)
Lack of clinical skill	3	(1.1)
Lack of motivation	3	(1.1)
Lack of qualified skill set	2	(0.7)
Lack of knowledge, clinical skill, and motivation	1	(0.4)
Poor attitude, knowledge, and skill	1	(0.4)
**Resources**	**90**	**(32.2)**
Lack of resources	67	(24.0)
Increased workload	23	(8.2)
**Organizational characteristics**	**69**	**(24.8)**
Policy, procedure, protocol	28	(10.1)
Organizational/system	18	(6.5)
Non‐compliance to policy and procedure	7	(2.5)
Poor documentation practices	6	(2.1)
Inadequate access	4	(1.4)
Organizational culture	3	(1.1)
Financial	3	(1.1)
**Total barriers:**	**279**	**100.0**

The most frequently identified barrier was a lack of resources (*n* = 67; 24%). A lack of assessment tools was the most reported lack of resource, followed by a lack of equipment, educational material, facilities, and services (e.g., acute pain service). The second most frequently reported barrier was a lack of knowledge (*n* = 64; 22%), with health professionals’ lack of knowledge reported as the most common knowledge barrier, followed by patients, patients and caregivers, political leaders, and caregivers.

### Congruence of Barriers to the Donabedian Dimensions of Care

Using the Donabedian approach to categorization, two independent assessors agreed on the SPO domains in 88% of the sample (*n* = 22/25). The *k* statistic for the pairwise coding was classed as excellent (*k* = .87; 95% CI [0.73, 1.0]).

For the descriptive data related to each of the barrier categories, overall agreement between the two assessors was obtained for 72.7% (*n* = 16/22) of the sample. Discrepancy in assigning categories was noted in six out of 22 (27.3%) observations. These results correspond to a *k* = .69, 95% CI [0.48, 0.91]; thus, the strength of agreement was good.

Table 3 shows the identified barriers mapped to Donabedian SPO structure, with the most frequently identified groupings of barriers being process‐related and associated predominantly with staff knowledge. Less frequently identified barriers were outcome‐related dimensions of care. The frequency calculation serves as an indicator of potential strength of the barrier as an obstacle to evidence implementation.

**Table 3:** Frequency of Findings for Each of the Barrier Categories

Barrier groupings/Donabedian categories	Frequency	%
**Structure**	**118**	**42.3**
Facilities and equipment	27	
Qualification of care providers	2	
Administration structure	10	
Operations of program	79	
**Process**	**156**	**55.9**
Communication	11	
Staff knowledge	68	
Patient knowledge	24	
Performance appraisal	10	
Quality of care	43	
**Outcome**	**5**	**1.8**
Organization and health System		3
Patient		2

### Implementation Strategies

Implementation strategies used in the different clinical projects were mapped against the EPOC taxonomy. In total, 565 strategies were used across all projects, with every project incorporating more than one strategy to address barriers to implementation of EBP. All four EPOC topics were represented: implementation strategies (*n* = 347; 61.4%); governance arrangements (*n* = 37; 6.5%); delivery arrangements (*n* = 174; 30.8%); and financial arrangements (*n* = 7; 1.3%). This representation allowed for the mapping of 11 (out of the 15 in total) sub‐categories. See Table 4 for complete mapping.

**Table 4:** EPOC Taxonomy (*n* = 565*)

EPOC topics	EPOC category	EPOC sub‐category	*n*	(%)
Implementation strategies	Interventions targeted at healthcare workers	Educational meetings	119	(21.1)
		Patient‐mediated interventions	83	(14.7)
		Monitoring the performance of the delivery of health care	42	(7.4)
		Educational materials	36	(6.4)
		Audit and feedback	10	(1.8)
		Local opinion leaders	10	(1.8)
		Routine patient‐reported outcome measures	8	(1.4)
		Tailored interventions	8	(1.4)
		Educational outreach visits or academic detailing	7	(1.2)
		Local consensus processes	6	(1.1)
		Continuous quality improvement	4	(0.7)
		Reminders	3	(0.6)
		Clinical practice guidelines	3	(0.5)
		Interprofessional education	2	(0.4)
		Reminders	1	(0.2)
		Academic detailing	1	(0.2)
		Educational games	1	(0.2)
		Managerial supervision	1	(0.2)
	Interventions targeted at healthcare organizations	Organizational culture	3	(0.5)
Governance arrangements	Authority and accountability for health professionals	Professional competence	25	(4.4)
		Authority and accountability for quality of practice	9	(1.6)
		Scope of practice	3	(0.5)
Delivery arrangements	Coordination of care and management of care processes	Care pathways	79	(14.0)
		Procurement and distribution of supplies	19	(3.4)
		Communication between providers	14	(2.5)
		Shared decision‐making	4	(0.7)
		Referral systems	3	(0.5)
		Teams	1	(0.2)
	Who provides care and how the healthcare workforce is managed	Staffing models	19	(3.4)
		Self‐management	9	(1.6)
		Role expansion or task shifting	2	(0.4)
	How and when care is delivered	Coordination of care among different provider	13	(2.3)
		Queuing strategies	1	(0.2)
		Triage	1	(0.2)
	Information and communication technology (ICT)	The use of information and communication technology	3	(0.5)
		Health information systems	2	(0.4)
	Where care is provided and changes to the healthcare environment	Environment	2	(0.4)
		Site of service delivery	2	(0.4)
Financial arrangements	Targeted financial incentives for health professionals and healthcare organizations	Pay for performance – target payments	3	(0.5)
	Mechanisms for payment of health services	Pricing and purchasing policies	2	(0.4)
	Collection of funds	External funding	2	(0.4)
**Total**			**565**	

* Multiple strategies per implementation report.

The vast majority of strategies (*n* = 119; 21.1%) were sub‐categorized as educational meetings for interventions targeted at healthcare workers. Patient‐mediated interventions (*n* = 83; 14.7%) were the second most identified strategy. The third most identified strategy was delivery of care pathways (*n* = 79; 14.0%).

## DISCUSSION

This paper reports the barriers encountered, and strategies utilized, for the implementation of EBP in LMICs. The approach to classification and evaluation against both the Donabedian framework and the EPOC taxonomy facilitated accurate reporting, indexing, and transparency in the analysis of barriers and the types of strategies used to address these barriers. Cultural and context‐specific issues—including but not limited to geography, language, limited resources, lack of technology, outdated infrastructure, and lack of access to available research—may hamper evidence implementation efforts. Therefore, a core set of strategies, alongside identified barriers to implementation, may prove useful in developing methods by which implementation programs can be implemented in LMICs (Lizarondo et al., 2019). Such a core list may be found useful in informing future implementation of evidence‐based interventions, a key obstacle in progressing the development of EBP in many LMICs (Puchalski Ritchie et al., 2016).

Included projects involved multi‐ and single‐disciplinary teams in acute, outpatient, and community settings. Most implementation projects were nurse‐led (*n* = 44; 73.3%) and occurred in acute care settings (*n* = 50; 83.3%), thus trended toward using more nurse‐led models of leadership. Previous research has highlighted that the role of nurse practitioners, whose work covers diagnostic activities through to intervention‐based treatments, is said to be shaped by their contextual surroundings more so than other healthcare professionals (Gardner et al., 2014).

Within the 60 reports conducted in LMICs over a 10‐year period, common barriers were noted to relate to knowledge, skills, and attitude; resources; and organizational characteristics, as shown in Table 2. When undertaking the implementation, barriers can make it difficult to fully implement evidence‐based recommendations for practice (Malik, McKenna, & Plummer, 2016). This project has provided a focused set of barriers aimed at LMICs that may be useful in informing future projects. Reporting these barriers, by classifying them to the Donabedian framework, has led to defining unique themes that identify common obstacles to evidence implementation in LMIC healthcare settings.

In LMICs, the ability to identify and respond to health care locally may be undermined by the lower research capacity and absence of clinical practice guidelines (Dean, Gregorius, Bates, & Pulford, 2017). All implementation strategies in the clinical fellowship program are based on the identification of barriers to achieve EBP for a specific population and setting. This evidence base comes from audit criteria identified by JBI that are sourced directly from a recommendation supported by the most current evidence. To define these intervention strategies in a useful and translatable way, all interventions were mapped to the EPOC framework and used as a structure for classifying the interventions while also providing a common terminology to describe numerous different settings (Johnson & May, 2015). In the context of describing interventions used to promote and integrate EBP into clinical care, inconsistency in terminology is a potential barrier to implementation (Colquhoun et al., 2014).

To be successful, interventions must be designed to reach the population of interest; however, most importantly, they must be based on a rigorous theoretical underpinning to explain why they may succeed or fail (Nilsen, 2015). Only when interventions are targeted specifically within the socio‐environmental setting for which they are intended will implementation succeed (Lizarondo et al., 2019; Ojo et al., 2019). This concept was supported by an umbrella review of 67 systematic reviews that found that the EPOC framework enabled different intervention types to be classified and mapped to common and effective interventions (Johnson & May, 2015). In our study, we found that the EPOC taxonomy provided a methodological vocabulary enabling a shared understanding of strategies across settings. This allowed for a common description of interventions used in vastly different settings (e.g., countries and participant groups) to be grouped into a commonly used list of domains.

Translating research into clinical practice remains considerably challenging (Breimaier et al., 2013; Johnson & May, 2015). Guidelines for healthcare decision‐making by clinicians and other healthcare professionals are continually updated as the evidence base changes, and regardless of geographic location or economic status, the demand for the rapid uptake of EBP by policymakers, healthcare providers, and health systems is universal (Dizon et al., 2017). However, despite this universality, health systems in LMICs are presented with greater challenges than those in high‐income countries; one of these differences is the availability of resources (Wiysonge et al., 2017). This was reflected in our data, where a common barrier to implementation was identified as a lack of resources with a high degree of commonality found across countries and clinical areas, with lack of tools, equipment, educational materials, facilities, and services most prominent. Strategies used to overcome these barriers were focused primarily on procurement and distribution of supplies, staffing models, and external funding; however, all projects used multiple strategies to address their barriers. Although these strategies were used to address the most common barrier, they were not the most commonly reported strategies in the implementation projects. This may indicate that participants felt that these issues were outside of their influence.

A second umbrella review (Pantoja et al., 2017), which assessed the effects of strategies for implementing interventions to improve health in low‐income countries, found that most of the available evidence was focused on strategies targeted at healthcare workers and healthcare recipients and related to process‐based outcomes. They reported that evidence of the effects of strategies targeting healthcare organizations was scarce (Pantoja et al., 2017). A qualitative review also reported the importance of understanding individual and organizational behaviors and motivation to support implementation in LMIC settings (Stokes et al., 2016). Results from these studies support what was found in our examination of the implementation projects, where the top strategies were strategies for educational meetings and patient‐mediated interventions targeted at healthcare workers and the delivery of care pathways for patients.

### Limitations

The current study is limited by the small number of LMICs represented in the implementation reports, with over half (56.7%) of these coming from China and a large portion from Africa (30%). Although many of the barriers across different LMICs may be similar, it is important to acknowledge that location, context, and cultural differences are possible, which may present further barriers to implementation. Additionally, only published implementation studies in the JBISRIR were included for analysis in this paper, which is not a true representation of all the EBCFPs undertaken.

## CONCLUSION

Translating healthcare evidence into practice continues to remain challenging (Jabbour, Newton, Johnson, & Curran, 2018). Knowledge synthesis has now emerged as an essential part of knowledge translation. The data presented here have shown essential considerations for a successful evidence‐based implementation program in LMICs. Our findings have provided an initial road map for undertaking implementation projects in LMICs, allowing project leads to anticipate barriers and construct evidence‐based strategies to overcome them. The use of the Donabedian framework to classify barriers, and the EPOC to classify strategies, has ensured a systematic approach in this process. As we look to the 2030 milestone of Sustainable Development Agenda for universal health coverage (World Health Organization, 2014), this commonality between barriers to implementation may be useful in the planning of future implementation projects in LMICs.

## RECOMMENDATIONS FOR PRACTICE

Translating healthcare evidence into practice remains challenging, especially in LMICs where resources may be scarce. The barriers and strategies identified in this project are likely to be similar across LMICs and should be considered when designing future evidence implementation projects. It has been shown that barriers are able to be addressed with minimal resource requirement, predominantly through education strategies targeted directly at healthcare workers.

## RECOMMENDATIONS FOR FUTURE RESEARCH

Future research should explore what features of an intervention are effective in one context and how this could be translated into another. There is also scope to examine similarities and differences between barriers and strategies from implementation studies undertaken in developed countries compared to those presented here for LMICs.

**Linking Evidence to Action**

​

- Context‐specific strategies and facilitatory methods are required for successful evidence implementation in LMICs.
- Common barriers can be addressed using locally available, low‐cost resources, for which multiple strategies are more common than single strategies.
- Education for healthcare workers in LMICs is an effective awareness‐raising, workplace culture, and practice‐transforming strategy for evidence implementation.
- Audit and feedback highlight many process‐driven aspects of care planning that can be improved by using the Donabedian SPO dimensions of care.
- Audit and feedback may be less useful for influencing patient outcomes and more useful for staff knowledge, skills, and care‐planning processes.
- The EPOC taxonomy may facilitate a shared understanding of implementation strategies across diverse settings, but the Donabedian SPO model has stronger, immediate clinical application in this context.

​

​

## CONFLICTS OF INTEREST

All authors are staff members or a visiting scholar (LF) at JBI, where the Evidence‐Based Clinical Fellowship program is conducted. LL is an educator and facilitator for the Evidence‐Based Clinical Fellowship program.

## Supporting information


**Table S1** GRiP (Getting Research into Practice) Barriers and Strategies for Evidence Implementation for Diabetes Self‐Management Education Training in Indonesia (Sugiharto, Stephenson, Hsu, & Fajriyah, 2017).
