# Supplementary material for: Mapping Clinical Barriers and Evidence‐Based Implementation Strategies in Low‐to‐Middle Income Countries (LMICs)
Source: Worldviews Evid Based Nurs. 2021 May 10;18(3):190–200. doi: 10.1111/wvn.12503 (PMC8251858; doi:10.1111/wvn.12503)
Supplement: Supplementary file 1 — Table S1 GRiP (Getting Research into Practice) Barriers and Strategies for Evidence Implementation for Diabetes Self‐Management Education Training in Indonesia (Sugiharto, Stephenson, Hsu, & Fajriyah, 2017). [file WVN-18-190-s001.docx]

**Table S1.** GRiP (Getting Research into Practice) Barriers and Strategies for Evidence Implementation for Diabetes Self-Management Education Training in Indonesia (Sugiharto, Stephenson, Hsu, & Fajriyah, 2017)

| **Target audience** | **Barrier** | **Strategies** | **Resources** | **Outcomes** |
| --- | --- | --- | --- | --- |
| Staff: | Diabetic education is not delivered properly  No education materials provided  Lack of knowledge and emphasis on diabetes self-management  Poor staff attitude toward DSME | Explain to staff about the project and the importance of DSME  Train PICs on DSME  Effective utilization of education materials for patient education | Evidence-based DSME resources  Evidence-based questionnaire on the level of confidence in carrying out diabetes education | Change of staff attitude  More knowledge and skills on DSM  PICs are more skilful at patient education |
| Administration: | Policy on DSME | Develop a new policy on DSME program  All PICs to be trained in DSM before they educate the patient | A new policy on DSME program  Training in DSME for PICs | Development of patient DSME program |
| Patients: | Lack of knowledge and awareness of self-management  Limited engagement by family members | Provide DSME  Empower family members | Patient education materials | More knowledge on self-management by patients and family members  Improvement of self- management  PICs competent in patient education |

*Note.* The JBI software PACES (Practical Application of Clinical Evidence System) and GRiP (Getting Research into Practice) audit and feedback tools were utilized in this project for promoting evidence utilization and changes in the community health setting. The team analyzed the baseline results for the 12 audit criteria and used the GRiP component of PACES to identify gaps and barriers related to the Diabetes Self-Management Education Program (Sugiharto, Stephenson, Hsu, & Fajriyah, 2017). The GRiP table highlights specific barriers and strategies in this context.
